# High patient acceptability but low coverage of provider-initiated HIV testing among adult outpatients with symptoms of acute infectious illness in coastal Kenya

**DOI:** 10.1371/journal.pone.0246444

**Published:** 2021-02-05

**Authors:** Clara A. Agutu, Tony H. Oduor, Bernadette K. Kombo, Peter M. Mugo, Salome M. Chira, Fred W. Ogada, Tobias F. Rinke de Wit, Wairimu Chege, Elise M. van der Elst, Susan M. Graham, Eduard J. Sanders

**Affiliations:** 1 Kenya Medical Research Institute-Wellcome Trust Research Programme, Kilifi, Kenya; 2 Department of Global Health, Academic Medical Centre, University of Amsterdam, Amsterdam, The Netherlands; 3 Prevention Sciences Program, Division of AIDS (DAIDS), National Institute of Allergy and Infectious Diseases (NIAID), National Institutes of Health (NIH), Rockville, Maryland, United States of America; 4 Departments of Global Health, Medicine, and Epidemiology, University of Washington, Seattle, Washington, United States of America; 5 Centre for Tropical Medicine and Global Health, Nuffield Department of Medicine, University of Oxford, Oxford, United Kingdom; University of the Witwatersrand, SOUTH AFRICA

## Abstract

**Background:**

Only approximately one in five adults are offered HIV testing by providers when seeking care for symptoms of acute illness in Sub-Saharan Africa. Our aims were to estimate testing coverage and identify predictors of provider-initiated testing and counselling (PITC) and barriers to PITC implementation in this population.

**Methods:**

We assessed HIV testing coverage among adult outpatients 18–39 years of age at four public and two private health facilities in coastal Kenya, during a 3- to 6-month surveillance period at each facility. A subset of patients who reported symptoms including fever, diarrhoea, fatigue, body aches, sore throat or genital ulcers were enrolled to complete a questionnaire independently of PITC offer. We assessed predictors of PITC in this population using generalised estimating equations and identified barriers to offering PITC through focus group discussion with healthcare workers (HCW) at each facility.

**Results:**

Overall PITC coverage was 13.7% (1600 of 11,637 adults tested), with 1.9% (30) testing positive. Among 1,374 participants enrolled due to symptoms, 378 (27.5%) were offered PITC and 352 (25.6%) were tested, of whom 3.7% (13) tested positive. Among participants offered HIV testing, 93.1% accepted it; among participants not offered testing, 92.8% would have taken an HIV test if offered. The odds of completed PITC were increased among older participants (adjusted odds ratio [aOR] 1.7, 95% confidence interval [CI] 1.4–2.1 for 30–39 years, relative to 18–24 years), men (aOR 1.3, 95% CI 1.1–1.7); casual labourers (aOR 1.3, 95% CI 1.0–1.7); those paying by cash (aOR 1.2, 95% CI 1.0–1.4) or insurance (aOR 3.0, 95% CI 1.5–5.8); participants with fever (aOR 1.5, 95% CI 1.2–1.8) or genital ulcers (aOR 4.0, 95% CI 2.7–6.0); and who had tested for HIV >1 year ago (aOR 1.4, 95% CI 1.0–2.0) or had never tested (aOR 2.2, 95% CI 1.5–3.1). Provider barriers to PITC implementation included lack of HCW knowledge and confidence implementing guidelines, limited capacity and health systems constraints.

**Conclusion:**

PITC coverage was low, though most patients would accept testing if offered. Missed opportunities to promote testing during care-seeking were common and innovative solutions are needed.

## Introduction

HIV testing remains the gateway to HIV prevention, treatment, care and other support services. To achieve universal test coverage, the World Health Organization (WHO) in 2007, recommended an “opt-out” testing approach known as Provider-Initiated HIV Testing and Counselling (PITC), in which an HIV test is offered to all patients attending health facilities in countries with generalised epidemics as a standard part of medical care [1]. However, a review looking at the operational implementation of PITC among non-pregnant adults in sub-Saharan (SSA) settings highlighted challenges in PITC implementation [2], with coverage estimated at only 20% [3]. PITC coverage has been lowest in general outpatient settings, despite a high yield of new HIV diagnosis [4–6]. High workload combined with high patient volume, personnel shortages, perceived slowing of patient flow, confusion about procedures, and operational challenges such as shortages of test commodities, inadequate infrastructure and space have been identified as barriers to implementing PITC in SSA [2, 7–9].

In 2018, Kenya had an estimated 1.4 million adults living with HIV [10]. National HIV prevalence was estimated at 4.9% in the same year, with an estimated 36,000 new adult infections [10]. Kenya has made progress towards achieving the UNAIDS “first 90” with an estimated 79.5% of people living with HIV (PLHIV) who knew their status in 2018 [10, 11], up from 53.0% in 2012 [12]. Initially, Kenya HIV testing and counseling (HTC) services were primarily provided through voluntary counseling and testing (VCT) sites where individuals who wanted to know their status could visit out of their own initiative [13]. From 2007, however, Kenya adopted multiple strategies to increase HIV test uptake, including routine PITC with targets set at 100% for inpatients and 50% for outpatient [13, 14]. Unfortunately, funding to support HTC counsellors, staffing and HIV tests has been limited and health worker attitudes have posed barriers to PITC scale-up [9, 13]. Population survey data in 2012 indicated that only a third (34%) of adults 15–64 years, visiting a health facility were offered an HIV test, despite high acceptability (91.5%) among those offered testing [15].

Routine HIV testing in Kenya has been implemented in antenatal care (ANC) settings [16, 17] and in tuberculosis (TB) clinics, where HIV screening has documented significant rates of co-infection [16, 17]. Missed opportunities for HIV testing however, remain in outpatient departments and other clinical settings [17]. Because current reporting tools aggregate facility-based HIV testing and do not allow for the estimation of PITC coverage by department [13, 18], it is difficult to compare PITC coverage across various clinical settings (e.g., outpatient, TB, ANC, VCT). Current national HIV testing service (HTS) guidelines in Kenya recommend annual HIV testing for the general population, to be carried out by routine testing in health facilities, VCT services, and other settings [19].

While young patients presenting for outpatient care with symptoms of acute illness may have higher rates of undiagnosed HIV infection [20–24], the rate at which they are offered PITC at care-seeking is not clear. Using data from the observational phase of a stepped-wedge trial of an HIV-1 RNA testing intervention that enrolled patients aged 18–39 years who sought urgent care for symptoms of acute infectious illness, we aimed to describe PITC coverage and factors associated with completed HIV testing in this population. In addition, we conducted focus group discussions with health care workers (HCW) in four public and two private health facilities in coastal Kenya to understand their general views on barriers to PITC implementation.

## Methods

### Study setting and population

The *Tambua Mapema* (“Discover Early” in Swahili) *Plus* Study (ClinicalTrials.gov Identifier: NCT03508908) is a proof-of-concept stepped-wedge trial assessing the impact of a health facility-based HIV testing intervention for detection of prevalent and acute HIV infection (AHI) using point-of-care HIV-1 RNA (Cepheid GeneXpert^®^ HIV-1 Qual) among young adult patients aged 18–39 years seeking care for symptoms compatible with acute infectious illness [25]. Between December 2017 and June 2019, the study recruited male and female adult patients presenting for care at four public and two private primary care facilities in Mombasa and Kilifi Counties (estimated adult HIV prevalence in 2018, 5.6% and 2.3%, respectively) [26]. Mombasa County is ranked ninth nationally out of 47 counties with respect to its HIV prevalence (5.6%) [26]. Kilifi County, just north of Mombasa County, includes several periurban, including Mtwapa, where a Kenya Medical Research Institute (KEMRI) Mtwapa research clinic has been operational since 2005. The six health facilities selected for this study, three in Mombasa County and three in Kilifi County, were chosen based on their proximity to the KEMRI research clinic in Mtwapa (within a 20-kilometer radius), ≥15 outpatient visits daily, and the availability of HIV testing services, including PITC. The facilities included a dispensary (1 public), 3 health centres (2 public, 1 private) and 2 hospitals (1 public, 1 private).

Eligibility criteria for the Tambua Mapema Plus (TMP) study included: 1) age 18–39 years; 2) not previously diagnosed with HIV infection; and 3) a score ≥2 on an AHI risk score algorithm with scoring as follows; age 18–29 years (1), fever (1), fatigue (1), body pains (1), diarrhoea (1), sore throat (1), and genital ulcer disease (GUD) (3) [24, 27]. Prior to implementing the testing intervention at each site, we observed facility HIV and malaria testing of patients presenting to the outpatient department and recorded the outcomes of facility-administered HTS among enrolled TMP study participants (i.e., the observation phase with 1,375 participants). PITC was offered according to national Kenyan HTS guidelines using rapid antibody HIV tests [19]. Linkage to care and treatment after HIV diagnosis was provided at the study facility or any other facility, as per participant preference and in accordance with national ART guidelines.

The current study employed an explanatory sequential mixed-method design [28] with focus group discussions (FGDs) conducted with providers working at the six health facilities following completion of quantitative data collection from the observation phase. FGDs were chosen to provide feedback on the quantitative findings and an opportunity for reflection on the challenges of PITC scale up experienced by facility staff [29]. Eligibility for the FGDs included age ≥ 18 years, employed for at least one year and planning to work at the facility for the duration of trial implementation there. The study protocol and detailed procedures have been published [25]. Data collection forms and the FGD topic guide were pilot tested and refined before implementation. The present analysis includes data collected during 3 months of observation period at the first site, followed by 6 months in each subsequent facility.

### Data collection procedures

For each study participant, data on age, symptoms, HIV testing history, and axillary temperature were collected by the research clinician or counsellor. Research staff (clinician or counsellors) obtained permission from the facility providers to be present during patient consultations for data collection. Patients with a risk score of ≥2 were offered enrolment by a study counsellor. Enrolled participants were administered a questionnaire collecting data including marital status, religion, education level and source of income. Sexual risk behaviour history over the past 6 weeks was obtained via computer-assisted self-interview (CASI). Additionally, we assessed days since symptoms began, mode of payment for services, and other costs to participants for their facility visit. From February 2018 (study period; December 2017 to June 2019), we also asked whether HIV testing was offered by the provider or requested by the participant, and for those not offered HIV testing, whether they would have agreed to an HIV test if requested by the provider. The outcomes of PITC and malaria tests ordered by the facility provider were captured.

As background information for each health facility, we extracted from routine facility testing registers, de-identified data on age, sex and HIV testing results, if done, on all outpatients aged 18–39 years registered in the same period that study patients were enrolled in the observational period of the study. Additionally, we collected data on facility attributes (including the type of facility and staffing), consultation fees, and available HIV testing and care services and malaria diagnostic services.

Following completion of the observation phase at each facility, and compilation of PITC data at each facility, a focus group discussion (FGD) was conducted at each health facility with between 8 to 11 facility staff members involved in the provision of HTS during that facility’s observation period. HCWs were purposively sampled to include representatives from different cadres including clinicians, nurses, HTS counsellors and laboratory technologists, to explore barriers to offering PITC. FGDs were conducted in English guided by a trained moderator. A semi-structured topic guide was used to explore their general experiences providing PITC and other HTS, to discuss which patients providers targeted for testing, and to solicit provider views on how to increase PITC completion rates. The findings of the quantitative analysis were integrated within the topic guide to give a more context specific discussion. Notes were taken by a second research team member. FGDs took up 2 hours and were recorded on a digital voice recorder following participants consent.

### Data analysis

To calculate facility HIV testing coverage among outpatients aged 18–39 years seeking primary care, we divided number of patients tested by the total number patients registered during the observation period. For enrolled participants, descriptive statistics were used to summarize frequencies and proportions for categorical data and medians and interquartile ranges for continuous data. The main outcome was PITC completed among study participants. Generalised estimating equations (GEE) regression with an exchangeable correlation matrix and robust standard errors was used to identify participant characteristics associated with completed PITC, taking into account clustering within health facility. Sensitivity analysis was conducted with PITC offer as an outcome. Covariates with a p value ≤0.10 in bivariable analysis were included in multivariable modelling; age and sex were included a priori. Associations with a p value ≤0.05 in multivariable modelling were considered significant. Data analysis was conducted using Stata ^®^ 15 (Stata Corp, USA).

Verbatim transcriptions were uploaded into NVivo software (Version 11 (2015), QSR International Pty Ltd) to assist in data management and analysis. Coding of transcripts used a thematic framework approach [30, 31]. A coding dictionary was developed from pre-determined parent codes based on the topic guide and themes related to barriers and facilitators to PITC implementation from literature and that emerged from summary notes compiled by the moderator and note-taker at the end of each FGD. To ensure reliability, data from the provider FGDs were triangulated with data from the quantitative analysis and facility testing registers. Excerpts from the FGDs presented in this paper were edited to aid readability.

### Ethical considerations

The study received ethical review and approval by the KEMRI Scientific and Ethical Review Unit (KEMRI/SERU/CGMRC-C/051/3280), the University of Washington Human Subjects Division (STUDY00001808) and the University of Oxford Tropical Research Ethics Committee (OxTREC) (Reference: 46–16). Written informed consent was obtained from all study participants.

## Results

### Health facility characteristics

Characteristics of the six primary care health facilities at which participants were enrolled are presented in Table 1. In the majority of the facilities, clinical services were provided by clinical officers and nurses. All facilities offered HIV testing and care services, with free HTS across all four public facilities. In the majority of facilities, following recommendation by the provider, HTS was performed by counsellors or laboratory technicians. The cost of PITC at the two private facilities was KSH 200 (US$ 2) and KSH 350 (US$ 3.5); this service was free at public facilities. Between December 2017 and June 2019, overall HIV testing coverage among adult patients (not all research participants) aged 18–39 years at these facilities was 13.7% (1,600 / 11,637). Of those tested, 1.9% (30) were newly diagnosed. Test coverage among patients in this age group ranged from 3.9%-37.0% among public health facilities and from 9.2%-20.6% among private facilities. HIV-positivity ranged from 1.0%-3.0% at public health facilities and from 2.4%-3.4% at private facilities. Additional information on health facility characteristics can be found in the supplemental materials (S1 Table).

**Table 1:** Characteristics of the six health facilities selected for participation in the Tambua Mapema Plus Trial, 2017–2020.

Characteristic	Health facility 1	Health facility 2	Health Facility 3	Health facility 4	Health facility 5	Health facility 6
**County**	Kilifi	Kilifi	Kilifi	Mombasa	Mombasa	Mombasa
**Facility operations**						
**Type of health facility**	Private	Private^1^	Public	Public	Public	Public
**Consultation cost**	KES 100	KES 400/450^2^	Free	KES 30	KES 20	KES 130
**Staff offering HTS**	Lab-techs, NOs, COs	VCT counsellors and Lab-techs	VCT counsellors, Lab-techs and COs	VCT counsellors, Lab-techs and NOs	VCT counsellors and Lab-techs	VCT counsellors and Lab-techs
**Cost of HIV tests**^3^	KES 200	KES 350	Free	Free	Free	Free
**Facility data during the study period**						
**Patient visits among adults 18–39 years registered at the facility**	1401	3207	2065	2051	1636	1277
**Testing encounters among adults 18–39 years tested for HIV, n (%)**^4^	288 (20.6)	294 (9.2)	764 (37.0)	79 (3.9)	108 (6.6)	67 (5.2)
**HIV positivity among testing encounters, n (%)**^4^	7 (2.4)	10 (3.4)	8 (1.0)	1 (1.3)	2 (1.9)	2 (3.0)

**Abbreviations**: CO: Clinical Officer, HTS: HIV testing and counselling services, Lab-tech: Laboratory technologist, NO: Nursing Officer/Nurse, VCT: Voluntary Counselling and Testing

Exchange rate: approximately 1 USD = 100 KES

^1^ Faith based organisation

^2^ Day charges -KES 400, night charges- KES 450

^3^ Cost of rapid antibody tests

^4^ Number inclusive of participants enrolled in the observation phase of the TMP study. A total of 11,637 patient visits among adults 18–39 years registered at the facility during the study period, with 1600 (13.7%) HIV testing encounters of which 30 (1.9%) resulted in a HIV diagnosis.

### Characteristics of study participants

Participant characteristics are presented in Table 2. Of 1,374 study participants enrolled between December 2017 and June 2019, mean age was 26 years (SD 4.8), 64.5% were female, 47.2% were single, 39.5% had secondary education, 76.6% were Christian and 51.5% were employed. The vast majority (95.9%) reported that their current symptoms had started ≤14 days ago; 65.8% reported fatigue, 64.5% body aches, 48.3% fever, 27.4% sore throat, 14.2% diarrhoea, and 6.6% genital ulcers. Overall, 494 (36.0%) of enrolled participants last tested for HIV >1 year ago and 147 (10.7%) had never tested for HIV; of these 133 (26.9%) and 59 (40.1%), respectively, had an HIV test requested by the provider and completed testing. A malaria test was requested for 48.6% of participants (of whom 64.7% had reported fever or had a temperature ≥ 37.5°Celsius). Overall, 378 participants (27.5%) were offered PITC and 352 (25.6%) completed testing.

**Table 2:** Factors associated with provider-initiated HIV testing and counselling (PITC) completion among study participants, n = 1,374.

Characteristics	Total, n (%)	Total PITC^1^, n (%)	Crude OR [95% CI]	P-Value	Adjusted OR [CI]	P-Value
**Type of facility**						
**Private**	374 (27.2)	46 (12.3)	[ref]	0.503		
**Public**	1000 (72.8)	306 (30.6)	1.82 (0.32, 10.43)			
**Age**						
**18–24 years**	644 (46.9)	167 (25.9)	[ref]		[ref]	
**25–29 years**	439 (32.0)	105 (23.9)	1.10 (0.89, 1.36)	0.379	1.23 (0.96, 1.57)	0.105
**30–39 years**	291 (21.2)	80 (27.5)	1.60 (1.33, 1.92)	<0.001	1.70 (1.38, 2.10)	<0.001
**Sex**						
**Female**	886 (64.5)	204 (23.0)	[ref]		[ref]	
**Male**	488 (35.5)	148 (30.3)	1.35 (1.22, 1.49)	<0.001	1.32 (1.05, 1.67)	0.019
**Marital status**^2^						
**Single**	649 (47.2)	180 (27.7)	[ref]			
**Married**	635 (46.2)	148 (23.3)	1.06 (0.77, 1.45)	0.722		
**Separated, divorced, widowed**	86 (6.3)	23 (26.7)	1.15 (0.62, 2.13)	0.667		
**Level of education**^2^						
**Primary and below**	513 (37.3)	158 (30.8)	[ref]		[ref]	
**Secondary**	542 (39.5)	129 (23.8)	0.73 (0.47, 1.12)	0.148	0.74 (0.43, 1.26)	0.267
**Higher education**	315 (22.9)	64 (20.3)	0.63 (0.40, 0.99)	0.045	0.64 (0.37, 1.10)	0.109
**Religion**^2^						
**Christians**	1053 (76.6)	263 (25.0)	[ref]			
**Muslims**	295 (21.5)	81 (27.5)	1.14 (0.97, 1.33)	0.111		
**None**	22 (1.6)	7 (31.8)	1.24 (0.67, 2.30)	0.485		
**Source of income**^2^						
**Employed**	707 (51.5)	162 (22.9)	[ref]		[ref]	
**Unemployed**	478 (34.8)	122 (25.5)	0.96 (0.88, 1.05)	0.397	1.12 (0.94, 1.34)	0.206
**Casual labourers**^3^	185 (13.5)	67 (36.2)	1.37 (1.18, 1.59)	<0.001	1.31 (1.02, 1.68)	0.035
**Payment for consultation or use of national social insurance card**						
**Free services**	360 (26.2)	73 (20.3)	[ref]		[ref]	
**Social insurance (NHIF**^4^**)**	301 (21.9)	41 (13.6)	1.16 (0.61, 2.19)	0.656	1.15 (0.54, 2.44)	0.710
**Cash**	700 (51.0)	236 (33.7)	1.13 (0.90, 1.42)	0.306	1.19 (1.04, 1.37)	0.011
**NHIF and cash**	6 (0.4)	1 (16.7)	0.50 (0.11, 2.15)	0.349	0.79 (0.19, 3.21)	0.740
**Other schemes**^5^	7 (0.5)	1 (14.3)	2.15 (1.25, 3.69)	0.005	2.98 (1.53, 5.82)	0.001
**Risk group**^6^						
**Sexually active general population**	897 (65.3)	215 (24.0)	1			
**Sexually active key populations**^7^	19 (1.4)	3 (15.8)	0.56 (0.23, 1.35)	0.197		
**Not sexually active, past 6 weeks**	458 (33.3)	134 (29.3)	1.00 (0.89, 1.12)	0.967		
**Time since last HIV test**						
**≤ 1 year ago**	733 (53.4)	160 (21.8)	[ref]		[ref]	
**More than 1 year ago**	494 (36.0)	133 (26.9)	1.40 (1.05, 1.86)	0.020	1.44 (1.03, 2.03)	0.033
**Never tested**	147 (10.7)	59 (40.1)	1.87 (1.43, 2.45)	<0.001	2.16 (1.51, 3.09)	<0.001
**Temperature**						
**<37.5**	1166 (84.9)	284 (24.4)	[ref]			
**> = 37.5**	208 (15.1)	68 (32.7)	1.36 (0.92, 2.03)	0.128		
**Reported fever**	663 (48.3)	195 (29.4)	1.27 (0.98, 1.65)	0.069	1.49 (1.23, 1.81)	<0.001
**Reported diarrhoea**	195 (14.2)	46 (23.6)	1.04 (0.79, 1.37)	0.768		
**Reported fatigue**	904 (65.8)	245 (27.1)	1.13 (0.89, 1.43)	0.333		
**Reported body aches**	886 (64.5)	233 (26.3)	0.96 (0.71, 1.30)	0.812		
**Reported sore throat**	376 (27.4)	95 (25.3)	1.11 (0.97, 1,27)	0.146		
**Reported genital ulcers**	91 (6.6)	47 (51.7)	2.94 (1.94, 4.45)	<0.001	4.04 (2.70, 6.04)	<0.001
**Days since symptoms began**^2^						
**≤ 14 days**	1317 (95.9)	335 (25.4)	0.72 (0.41, 1.28)	0.264		
**Malaria test done**	668 (48.6)	222 (33.2)	1.51 (0.76, 3.02)	0.242		

OR = odds ratio

A priori factors (age and sex) and variables with a p value of ≤0.10 in bivariable analysis were included in the multivariable GEE model, which accounted for clustering by health facility.

^1^ Out of 1,374 enrolled participants, 25.6% (352) were tested for HIV. 3.1% (43) participants were not included in the regression analysis as it was unknown if the provider had offered HIV testing.

^2^ Data were missing for 4 participants due to corrupted Audio Computer-Assisted Self-Interview (ACASI) entries.

^3^ Casual labourers included those reporting daily or weekly wages.

^4^ National Health Insurance Fund

^5^ Includes payments made by company medical insurance or private insurance schemes

^6^ Risk group assessed only in those who reported to be sexually active in the past six weeks (n = 916)

^7^ Key populations include men who have sex with men (MSM) (n = 4), sex workers (n = 15) and people who inject drugs (PWID) (n = 1)

### Factors associated with PITC completion

In bivariable modelling, increased odds of PITC completion among participants was associated with older age (30–39 years vs. 18–29 years), male sex, casual labourers, payment using insurance, reported fever or genital ulcers, and testing >1 year ago or never having tested before (Table 2). Higher education was associated with decreased odds of PITC completion. In the multivariable model, older individuals (adjusted odds ratio [aOR] 1.7, 95% confidence interval [CI] 1.4–2.1 for 30–39 years, relative to 18–24 years), men (aOR 1.3, 95% CI 1.1–1.7); casual labourers (aOR 1.3, 95% CI 1.0–1.7, relative to the employed); those paying by cash (aOR 1.2, 95% CI 1.0–1.4) or insurance (aOR 3.0, 95% CI 1.5–5.8), relative to free services); participants with reported fever (aOR 1.5, 95% CI 1.2–1.8) or genital ulcers (aOR 4.0, 95% CI 2.7–6.0); and participants who had tested for HIV >1 year ago (aOR 1.4, 95% CI 1.0–2.0) or had never tested (aOR 2.2, 95% CI 1.5–3.1, relative to testing in the past year) had higher rates of completed PITC. Sensitivity analysis on factors associated with PITC offer are presented in the supplemental materials (S2 Table).

### PITC outcomes and HIV positivity among study participants

**Fig 1:**
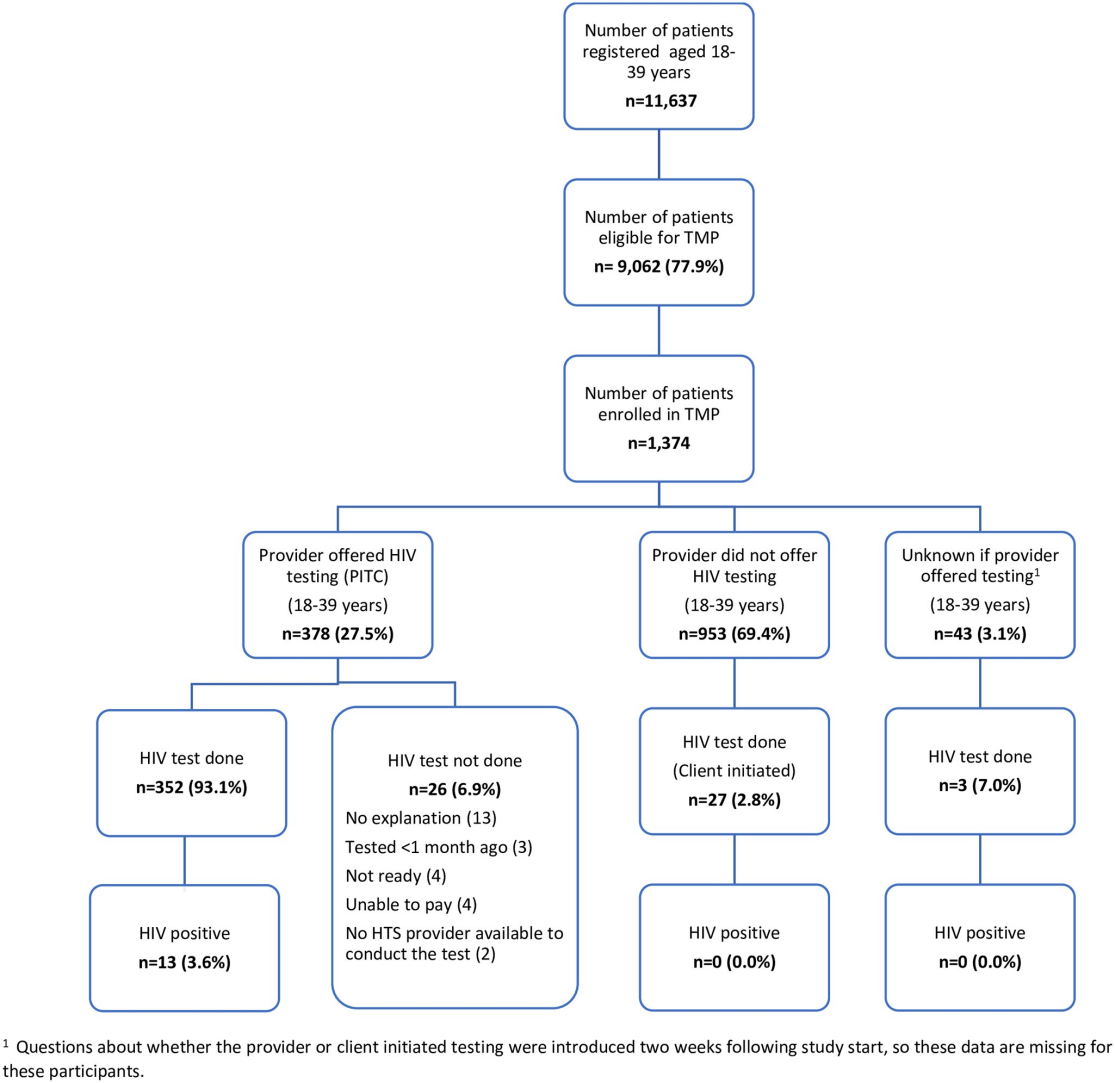
Study recruitment and outcome of provider-initiated HIV testing and counseling.

Fig 1 presents a flow diagram of study participants and testing outcomes. Among the 352 participants tested, 13 (3.7%) were diagnosed with HIV and all linked to HIV care at the facility of enrolment. Among participants not offered HIV testing by the provider, 2.8% (27) initiated HIV testing on their own. In addition, 43 (3.1%) of participants did not have clear documentation on whether the provider offered HIV testing.

PITC acceptance was the same among both men and women (93.0% for each). Twenty-four participants (6.3%) refused HIV testing for the following reasons: no explanation (13), tested <1 month ago (3), not ready (4), and unable to pay (4). For two patients, no HTS provider was available to conduct the test (Fig 1). Among participants not offered testing for HIV, 775 (81.3%) were asked a question on acceptability of HIV testing, which was added after the study began. Of these, 719 (92.8%) reported they would have tested for HIV if requested by the provider. Testing coverage (3.2%-56.4%) and HIV positivity (0.0%-8.3%) varied across facilities (Table 3).

**Table 3:** PITC coverage among adult research participants 18–39 years seeking primary care for symptoms of acute infectious illness at 6 health facilities, coastal Kenya 2017–2019.

Facility no.	Facility type	County	Facility testing among research participants (n = 1,374)		
			Patients 18–39 years enrolled	Patients 18–39 years tested for HIV, n (%)	HIV positivity among those tested, n (%)
**1**	Private	Kilifi	125	35 (28.0)	0 (0.0)
**2**	Private	Kilifi	249	11 (4.4)	1 (9.1)
**3**	Public	Kilifi	250	141 (56.4)	2 (1.4)
**4**	Public	Mombasa	250	8 (3.2)	0 (0.0)
**5**	Public	Mombasa	250	60 (24.0)	5 (8.3)
**6**	Public	Mombasa	250	97 (38.8)	5 (5.2)^1^
		**Total**	**1374**	**352 (25.6)**	**13 (3.7)**

^1^ One participant had serodiscordant results for which the outcome of repeat testing is unknown

### Provider experiences and perceptions of PITC

Six FGDs were conducted with a total of 57 clinic staff (8 to 11 participants per FGD) (details in S3 Table). Three major themes emerged in the FGD.

#### Knowledge and confidence in implementing guidelines was lacking

Providers exhibited varying levels of knowledge of what PITC entailed, with some unaware of PITC and others who felt that HIV testing was a personal decision.

“Is there a policy that anybody who comes to the facility is tested?”

(38-year-old, male, pharmaceutical technologist, Health facility 4, public)

Some providers exhibited uncertainty about PITC procedures with one provider emphasizing the need for pre-test counselling and insisting that testing should not be done if the patient was not counselled first, contrary to current guidelines.

“I think according to the Kenyan algorithm, they say no patient should be tested without counselling, that is why each time a patient comes, they must go to the consultation room then lab.”

(31-year-old, male, laboratory technologist, Health facility 2, private)

There were mixed opinions on when retesting should be recommended to the general population. Some providers mentioned not offering HIV testing to those who reported their last test was in the past 6 months; others advised retesting every 3 months to patients suspected to have recent HIV exposure. Some providers recommended annual HIV testing even in the absence of sexual exposure; however, this was not consistent across the facilities.

“There are some instances like maybe the client has not completed 3months, or the last test was carried out less than 3, or maybe 6 months ago. There are some questions you ask the client for screening; is this client eligible even if he has been tested, you don’t just test because he has been sent.”

(36-year-old, female, counsellor, Health facility 5, public)

Providers commented on the challenges of lack of documentation of testing history and the reliance on patient self-report of recent testing.

*“…we don’t have a book that shows someone has tested, so most of them [the patients] say they just tested recently…there is no history that we look at, they just say they did it [HIV testing]”*.

(30-year old, female, medical officer, Health facility 2, private)

#### Given limited capacity, HIV testing is often targeted

Most providers reported targeting patients for HIV testing based on clinical signs and symptoms of advanced HIV disease such as symptoms of TB, Kaposi’s sarcoma, and other WHO stage 3 or 4 AIDS-defining illnesses. Those presenting to care with recurrent sexually transmitted infections (STI) and recurrent illnesses without an apparent diagnosis were also sent for HIV testing.

“…. There are those signs that you check for, looking at the patient, depending on your experience, there are those signs that will always be there in someone who is HIV positive…. there is that aspect of staging…, stage three and four, you can hardly miss that.”

(36-year-old, male, nurse, Health facility 1, private)

Some providers assessed history of recent exposure or targeted populations thought to be at risk, including those suspected to be sex workers.

“…There are female sex workers. they are normally very honest…like I said, we send them for testing…”

(30-year-old, female, clinical officer, Health facility 2, private)

In one facility, a risk assessment tool provided by an implementing partner was available for screening prior to testing; however, providers were not trained on how to administer it, and it was not uniformly employed.

“In some rooms we are provided with tools for screening, even at the pharmacy, and at the OPD [outpatient department]. We know the clinicians, there is a tool that was given by ‘Afya Pwani’ [U.S. Agency for International Development]; you ask them when was the last time you were tested, such questions…”

(34-year-old, female, acting medical superintendent, Health facility 6, public)

Priority for HIV testing was reported by some to be given to the TB clinic; women seeking ANC, postnatal, or family planning services; and child welfare clinics. Across all the facilities, providers felt that HIV testing was of highest priority for pregnant women, due to consequences of transmission to the unborn child; in general, providers demonstrated familiarity with Kenyan prevention of mother-to-child HIV transmission guidelines.

“Okay, sometimes back there was a case whereby we had some shortages of the Determine [HIV] test kits, so you give priority especially to the pregnant mothers who would even expose an extra party, as in maybe the baby….”

(40-year-old, female, nurse, Health facility 5, public)

#### Health systems factors constrained PITC offering

High patient volume, often combined with shortage of personnel including VCT and HTS counsellors, was mentioned by providers as barrier to routine offer of HIV testing. Patient queues were often long, such that providers prioritized quantity over quality.

“… on a particular day, we have a staffing issue, we have one clinician, at the lab we have one person, then the queue has come this far….the clinician asks about HIV and what have you, he/she writes and forwards, when they get to the lab, the issue is the same, a single person…. it is just clearing the queues, so that at least they [the patients] move because they are quarrelling at the outpatient, yeah.”

(37-year-old, female, HTS counsellor, Health facility 3, public)

Availability of space for conducting HIV testing within outpatient departments was noted as a challenge in all facilities. This was felt to greatly affect patient confidentiality, especially in settings with shared consultation rooms.

“…I would say space, we are space constraint. We do not have enough room for all our counsellors.”

(49-year-old, female, nurse-in-charge, Health facility 5, public)

In the private facilities, the cost of HIV testing was often a deterrent to testing, especially when patients had to pay for other lab tests ordered by providers.

“On a busy day, this patient has been here, he has queued to see you [HCW]…, apart from wanting them to do an HIV test, there are other tests that you require him to do. When you look at the cost, he tells you “I can’t afford the other tests”, so most of the times, I omit the HIV and I send them for the rest [of the tests]… because they can’t pay for it…”

(30-year-old female, clinical officer, Health facility 2, private)

Providers also reported limited training opportunities, which impacted negatively on their HIV testing services, especially when they missed training when HTS guidelines were updated.

“…. sometimes you find that when they are going for training, they [fellow HCW] are just allocated maybe one slot per facility and of course we are a number of testers…you are trained, then you have to come and give feedback, yet you cannot give everything because this was a seminar for five days with a lot of content covered, you cannot divulge everything that you learned….So, if there was a way to have trainings done for all the staff that will be better.”

(36-year-old, male, nurse, Health facility 1, private)

Some providers placed more emphasis on the achievement of testing targets than on referring all qualifying patients for PITC. HTS counsellors reported having their own testing targets, with a maximum of 15 patients in any HIV testing service delivery point to be tested per day per counsellor.

“Those [HTS counsellors] who are testing are hitting their targets, as in 15 clients in a day, which is 100% according to us.”

(45-year-olf, female, HTS counsellor, Health facility 6, public)

## Discussion

Our results describe HIV test coverage among adults aged 18–39 years who sought primary care at six health facilities in coastal Kenya. We found that PITC was offered to only 1 in 7 patients aged 18–39 years who sought care for any reason. Among study participants aged 18–39 years, all of whom sought care for symptoms compatible with an acute infectious illness, less than 1 in 4 were offered an HIV test by the facility provider. Our findings demonstrate multiple missed opportunities for HIV testing in primary care facilities on the Kenyan coast, similar to our earlier study [21] and findings in other settings in Africa [32–34]. There was stark variation of PITC coverage between facilities, likely due to varying levels of HIV programming support, including supply of commodities, training, and staffing of HTS providers as well as differences in reporting on different sources of HTS that made it difficult to evaluate PITC coverage. In private facilities, the cost of HIV testing may have impacted provider offer of testing and patient ability to pay for the service. Additional factors such as differences in clinic staffing, supervision, management and procedures may have also contributed to heterogeneity in test coverage across sites. The higher test coverage among study participants may have been due to their reported symptoms, which were required for study eligibility, or to characteristics of the non-research participants and their reasons for seeking care.

When study participants were offered PITC, 93.1% accepted it. Interestingly, amongst those not offered HIV testing, a similar proportion (92.8%) reported they would have tested for HIV if requested by the provider. This suggests that non-patient barriers may be the primary reason for low PITC rates. Indeed, we identified a number of barriers to offering routine HIV testing, including a low level of knowledge about current HTS guidelines and retesting recommendations, shortages of trained staff, inadequate testing space, and frequent outages of test kits. With limited supplies, testing was often prioritised for pregnant women and TB patients. Of note, providers did report offering diagnostic HIV testing to those with obvious signs and symptoms of advanced HIV.

Our quantitative findings showed that providers initiated HIV testing more often for patients who were older (>30 years), male, casual labourers, those who paid cash for their consultation or had insurance or other medical coverage, had a fever or genital ulcers, or had tested >1 year ago or had never before tested for HIV. Targeting those whose costs could be recovered through insurance schemes may reflect a facility concern with recouping of running costs. In Kenya, as in other parts of Africa, men often test late for HIV and initiate treatment at a more advanced stage of disease [35]. For example, in a 2017 report on the Kenyan epidemic, fewer young men than women in the 20–24 age range had tested for HIV (67.3% vs. 91.6%, respectively) [36]. Similarly, in the most recent national population-based HIV impact survey conducted in 2018, 72.6% of men vs 82.7% of women who tested positive were aware of their HIV status [10]. In our study, providers may have targeted men for PITC because they were aware of this disparity and knew that young women are frequently tested within ANC settings [35]. It is also notable that providers were more likely to test for HIV participants who reported genital ulcer disease or fever. Due to the known association between sexually transmitted infections (STIs) and HIV, national HTS guidelines emphasize the importance of testing persons with an STI and retesting them 4 weeks later [19]. From the qualitative and quantitative data, it was clear that providers were more likely to test individuals with a presentation compatible with a STI or with untreated or advanced HIV infection.

There is a large PITC implementation gap in Kenya, as in other countries of Sub-Saharan Africa (SSA), where overall PITC coverage has been estimated at only 20% [3]. In our study, over one third of participants had not tested for HIV within the last 12 months and 10.7% reported never having tested. It is therefore encouraging that such patients were more likely to be offered PITC by providers in our study. The WHO emphasizes annual HIV testing for sexually active individuals in settings with a high HIV burden (greater than 5% HIV prevalence), with more frequent retesting based on individual risks; Kenyan national HTS guidelines align with this WHO recommendation [19, 37]. Interestingly, in this study of patients with symptoms of acute infectious illness, providers offered HIV testing to those reporting fever, however they were more likely to test for malaria compared to HIV (48.6% vs 25.6%), indicating they may be more concerned about missing a malaria diagnosis than an HIV diagnosis, despite overall declining trends in malaria prevalence in the region, especially among adults [38]. HCW training on recognizing symptoms of acute HIV in the context of care seeking is needed, especially given the need to rule out acute HIV infection prior to pre- or post-exposure prophylaxis initiation [19, 39].

In our FGD, several challenges were identified by providers, including that HTS guidelines were not clearly understood and staff wanted additional training. While educating providers about HTS guidelines and about the consequences of late HIV diagnosis could help increase PITC coverage [40], PITC scale-up likely requires the removal of structural barriers faced by providers and patients [41, 42]. Initiating HIV testing before or during consultation has been shown to improve PITC coverage in OPD settings in SSA [6, 43]. In addition, HIV self-testing (HIVST) is an innovative, low-cost intervention that could help achieve the “first 90” [44] and improve the efficiency of facility-based testing services by reducing provider burden and patient wait times [45]. For instance, HIVST could be done while waiting for a malaria test result, and the same laboratory technician could document both results. HIVST has been demonstrated to increase testing by partners of women in ANC programs in Kenya [46, 47], and led to an increase in PITC coverage in a randomized trial in Malawi [45]. While other studies have cited poor referral systems for linkage to care and treatment after diagnosis as barriers to PITC implementation [2], this was not a concern in our study, in which all newly diagnosed study participants were promptly linked to care at the same facility.

Although there is a clear public health case for universal testing for HIV, widespread adoption and implementation is required [17, 48]. In the face of poor HIV test coverage across most regions of SSA, new strategies have to be identified to reach the “first 90” and eventually identify the “first 95%” [49]. Targeted testing may be beneficial, in light of flat or reduced funding and the need for greater efficiency in testing. Targeted testing may also be beneficial in areas of declining HIV prevalence. In settings of low HIV prevalence, despite an increase in HIV testing rate, universal testing did not improve HIV case detection in outpatient departments compared to the routine use of symptom-based diagnostic HIV testing [50, 51]. In an analysis of data from PEPFAR-funded HTC programmes between July 2017 and June 2018, De Cock et al found the highest yield of new HIV diagnosis arose from TB clinics at 9.6%, while yield was only 1.4% in over 13 million tests performed, of which 77% were categorized as PITC [17]. In our study, yield was slightly higher at 3.7% in the research participants tested (compared to 1.9% overall among patients in this age group presenting to the study facilities), suggesting that targeting testing of patients with symptoms suggestive of acute infectious illness may be a strategy that could increase the yield of testing and improve the efficiency of PITC in clinical settings.

Our study had some limitations. First, we looked at predictors of PITC amongst patients selected because they met an AHI symptom score and did not collect detailed data on patients who did not meet these criteria. Second, overall PITC coverage at each health facility was calculated from facility registry data that may be inaccurate and did not distinguish between our research participants and other patients. Third, while we did not order HIV testing for study participants, the presence of research team members in the clinic may have influenced offering of PITC to study participants. Finally, the study was conducted in only six health facilities in a limited area of coastal Kenya. Therefore, results may not be generalizable to all health facilities in the region. Despite these limitations, our study provided both quantitative and qualitative insights to PITC implementation.

## Conclusions

Our study showed missed opportunities for HIV testing among young adults aged 18–39 with symptoms of acute infectious illness presenting to primary care, despite higher HIV testing yield among those tested relative to yield among all patients in this age group who sought care for whatever reason. In Kenya, where most HIV testing is facility-based, PITC remains a key strategy for early identification and linkage to care of HIV-positive individuals. While we found that most patients would be willing to accept HIV testing if offered, facility staff faced numerous barriers to successfully increasing PITC rates. Prioritising patients who have not tested recently and those with symptoms compatible with acute infectious illness or STI-related symptoms may increase the efficiency of HIV testing, if validated in other settings.

## Supporting information

S1 Table Characteristics of the six health facilities selected for participation in the Tambua Mapema Plus Trial, 2017–2020. (DOCX)

S2 Table Factors associated with provider-initiated HIV testing and counselling (PITC) offer among study participants, n = 1,374. (DOCX)

S3 Table Characteristics of healthcare workers in provider focused group discussions from 6 health care facilities, coastal Kenya 2017–2019 (n = 57). (DOCX)
