# Supplementary material for: High patient acceptability but low coverage of provider-initiated HIV testing among adult outpatients with symptoms of acute infectious illness in coastal Kenya
Source: PLoS One. 2021 Feb 5;16(2):e0246444. doi: 10.1371/journal.pone.0246444 (PMC7864413; doi:10.1371/journal.pone.0246444)
Supplement: S1 Table — (DOCX) [file pone.0246444.s001.docx]

**S1 Table. Characteristics of the six health facilities selected for participation in the Tambua Mapema Plus Trial, 2017-2020**

| Characteristic | | Health facility 1 | | Health facility 2 | | Health facility 3 | | Health facility 4 | | Health facility 5 | | Health facility 6 | |
| --- | --- | --- | --- | --- | --- | --- | --- | --- | --- | --- | --- | --- | --- |
| Observation period duration^1^ | December 2017-February 2018 | | December 2017- May 2018 | | March- July 2018 | | August- December 2018 | | September 2018- March 2019 | | March- June 2019 | |  |
| Facility operations |  | |  | |  | |  | |  | |  | |  |
| Number of staff | 9  (2 CO, 5 NO, 2 lab techs) | | 29  (1 MO, 4 CO, 12 NO, 4 lab techs, 4 pharm techs, 4  VCT counsellors) | | 25  (4 CO, 12 NO, 4 lab techs, 1 pharmacy tech, 4 VCT counsellors) | | 26  (4 CO, 1 DO, 14 NO, 2 lab techs, 2 pharm techs, 3 VCT counsellors) | | 15  (1 CO, 7 NO, 2 lab techs, 2 pharm techs, 3 VCT counsellors) | | 62  (9 MO, 10 CO, 30 NO, 6 lab techs, 4 pharmacists, 1 pharm tech, 2 VCT counsellors) | |  |
| Venues for HIV testing | Laboratory and VCT | | Laboratory, VCT, ANC, maternity, and ward | | Laboratory, VCT, ANC, maternity, ward, well-baby and FP | | VCT, ANC, ward and well-baby clinic | | Laboratory, VCT, ANC and FP | | VCT, ANC and FP | |  |
| Cost of malaria test^2, 3^ | KES 150 | | KES 250/150 | | Free | | Free | | KES 50 | | KES 50 | |  |

**Abbreviations:** ANC: Antenatal care, CO: Clinical Officer, DO: Dental Officer, FP: family planning, Lab tech: Laboratory technologist, MO: Medical Officer, NO: Nursing Officer/Nurse, Pharm tech: Pharmaceutical Technologist, VCT: Voluntary Counselling and Testing

^1^ Predetermined observation period. Target recruitment set at 250 participants per facility over a 3-6 month period following a modified stepped-wedged design with the exception of the first site (health facility 1; 125 participants) having only 3 months of observation to allow for site preparations, staff training and study oversight.

^2^ Exchange rate: approximately 1 USD = 100 KES

^3^ Cost of microscopy/ cost of malaria rapid diagnostic test
