# Supplementary material for: High patient acceptability but low coverage of provider-initiated HIV testing among adult outpatients with symptoms of acute infectious illness in coastal Kenya
Source: PLoS One. 2021 Feb 5;16(2):e0246444. doi: 10.1371/journal.pone.0246444 (PMC7864413; doi:10.1371/journal.pone.0246444)
Supplement: S2 Table — (DOCX) [file pone.0246444.s002.docx]

**S2 Table. Factors associated with provider-initiated HIV testing and counselling (PITC) offer among study participants, n=1,374**

| Characteristics | | Total,  n (%) | Total PITC^1^,  n (%) | Crude OR [95% CI] | P-Value | Adjusted OR [CI] | | P-Value |
| --- | --- | --- | --- | --- | --- | --- | --- | --- |
|  | |  |  |  |  |  |  | |
| Type of facility | |  |  |  |  |  |  | |
| Private | | 374 (27.2) | 53 (14.2) | [ ref ] | 0.577 |  |  | |
| Public | 1000 (72.8) | | 325 (32.5) | 1.66 (0.28, 9.98) |  |  |  | |
|  | |  |  |  |  |  |  | |
| Age | |  |  |  |  |  |  | |
| 18-24 years | | 644 (46.9) | 180 (28.0) | [ ref ] |  | [ ref ] |  | |
| 25-29 years | | 439 (32.0) | 113 (25.7) | 1.09 (0.89, 1.33) | 0.416 | 1.23 (1.00, 1.50) | 0.05 | |
| 30-39 years | | 291 (21.2) | 85 (29.2) | 1.57 (1.36, 1.81) | <0.001 | 1.70 (1.49, 1.95) | <0.001 | |
|  | |  |  |  |  |  |  | |
| Sex | |  |  |  |  |  |  | |
| Female | | 886 (64.5) | 219 (24.7) | [ ref ] |  | [ ref ] |  | |
| Male | | 488 (35.5) | 159 (32.6) | 1.36 (1.19, 1.56) | <0.001 | 1.38 (1.03, 1.83) | 0.029 | |
|  | |  |  |  |  |  |  | |
| Marital status^2^ | |  |  |  |  |  |  | |
| Single | | 649 (47.2) | 195 (30.1) | [ ref ] |  |  |  | |
| Married | | 635 (46.2) | 158 (24.9) | 1.03 (0.81, 1.31) | 0.807 |  |  | |
| Separated, divorced, widowed | | 86 (6.3) | 24 (27.9) | 1.08 (0.56, 2.07) | 0.814 |  |  | |
|  | |  |  |  |  |  |  | |
| Level of education^2^ | |  |  |  |  |  |  | |
| Primary and below | | 513 (37.3) | 165 (32.2) | [ ref ] |  | [ ref ] |  | |
| Secondary | | 542 (39.5) | 141 (26.0) | 0.77 (0.52, 1.15) | 0.201 | 0.76 (0.46, 1.24) | 0.268 | |
| Higher education | | 315 (22.9) | 71 (22.5) | 0.68 (0.47, 0.98) | 0.039 | 0.67 (0.42, 1.10) | 0.112 | |
|  | |  |  |  |  |  |  | |
| Religion^2^ | |  |  |  |  |  |  | |
| Christians | | 1053 (76.6) | 279 (26.5) | [ ref ] |  | [ ref ] |  | |
| Muslims | | 295 (21.5) | 91 (30.9) | 1.25 (1.05, 1.49) | 0.013 | 1.14 (1.00, 1.29) | 0.046 | |
| None | | 22 (1.6) | 7 (31.8) | 1.14 (0.61, 2.13) | 0.680 | 0.96 (0.43, 2.13) | 0.925 | |
|  | |  |  |  |  |  |  | |
| Source of income^2^ | |  |  |  |  |  |  | |
| Employed | | 707 (51.5) | 175 (24.8) | [ ref ] |  | [ ref ] |  | |
| Unemployed | | 478 (34.8) | 133 (27.8) | 0.99 (0.88, 1.11) | 0.850 | 1.14 (0.92, 1.42) | 0.239 | |
| Casual labourers^3^ | | 185 (13.5) | 69 (37.3) | 1.30 (1.17, 1.45) | <0.001 | 1.24 (1.02, 1.51) | 0.027 | |
|  | |  |  |  |  |  |  | |
| Payment for consultation or use of national social insurance card | |  |  |  |  |  |  | |
| Free services | | 360 (26.2) | 77 (21.4) | [ ref ] |  | [ ref ] |  | |
| Social insurance (NHIF^4^) | | 301 (21.9) | 45 (15.0) | 0.99 (0.63, 1.55) | 0.967 | 0.94 (0.56, 1.58) | 0.816 | |
| Cash | | 700 (51.0) | 253 (36.1) | 1.13 (0.87, 1.46) | 0.362 | 1.17 (1.04, 1.32) | 0.008 | |
| NHIF and cash | | 6 (0.4) | 2 (33.3) | 1.13 (0.20, 6.48) | 0.889 | 1.78 (0.28, 11.22) | 0.537 | |
| Other schemes^5^ | | 7 (0.5) | 1 (14.3) | 1.81 (1.18, 2.78) | 0.007 | 2.27 (1.34, 3.85) | 0.002 | |
|  | |  |  |  |  |  |  | |
| Risk group^6^ | |  |  |  |  |  |  | |
| Sexually active general population | | 897 (65.3) | 231 (25.8) | 1 |  |  |  | |
| Sexually active key populations^7^ | | 19 (1.4) | 3 (15.8) | 0.50 (0.21, 1.21) | 0.124 |  |  | |
| Not sexually active, past 6 weeks | | 458 (33.3) | 144 (31.4) | 1.01 (0.91, 1.11) | 0.894 |  |  | |
|  | |  |  |  |  |  |  | |
| Time since last HIV test | |  |  |  |  |  |  | |
| ≤ 1 year ago | | 733 (53.4) | 169 (23.1) | [ ref ] |  | [ ref ] |  | |
| More than 1 year ago | | 494 (36.0) | 146 (29.6) | 1.50 (1.12, 2.00) | 0.006 | 1.54 (1.12, 2.11) | 0.007 | |
| Never tested | | 147 (10.7) | 63 (42.9) | 1.96 (1.38, 2.77) | <0.001 | 2.21 (1.51, 3.25) | <0.001 | |
|  | |  |  |  |  |  |  | |
| Temperature | |  |  |  |  |  |  | |
| <37.5 | | 1166 (84.9) | 304 (26.1) | [ ref ] |  |  |  | |
| >=37.5 | | 208 (15.1) | 74 (35.6) | 1.42 (0.87, 2.30) | 0.162 |  |  | |
|  | |  |  |  |  |  |  | |
| Reported fever | | 663 (48.3) | 206 (31.1) | 1.22 (0.93, 1.60) | 0.158 |  |  | |
|  | |  |  |  |  |  |  | |
| Reported diarrhoea | | 195 (14.2) | 49 (25.1) | 1.03 (0.74, 1.43) | 0.881 |  |  | |
|  | |  |  |  |  |  |  | |
| Reported fatigue | | 904 (65.8) | 264 (29.2) | 1.15 (0.87, 1.51) | 0.322 |  |  | |
|  | |  |  |  |  |  |  | |
| Reported body aches | | 886 (64.5) | 245 (27.7) | 0.89 (0.61, 1.30) | 0.535 |  |  | |
|  | |  |  |  |  |  |  | |
| Reported sore throat | | 376 (27.4) | 103 (27.4) | 1.13 (0.96, 1.32) | 0.131 |  |  | |
|  | |  |  |  |  |  |  | |
| Reported genital ulcers | | 91 (6.6) | 49 (53.9) | 2.86 (1.79, 4.56) | <0.001 | 3.30 (2.01, 5.42) | <0.001 | |
|  | |  |  |  |  |  |  | |
| Days since symptoms began^2^ | |  |  |  |  |  |  | |
| ≤ 14 days | | 1317 (95.9) | 360 (27.3) | 0.74 (0.39, 1.41) | 0.355 |  |  | |
|  | |  |  |  |  |  |  | |
| Malaria test done | | 668 (48.6) | 239 (35.8) | 1.54 (0.77, 3.09) | 0.224 |  |  | |

OR= odds ratio

A priori factors (age and sex) and variables with a p value of ≤0.10 in bivariable analysis were included in the multivariable GEE model, which accounted for clustering by health facility.

^1^ Out of 1,374 enrolled participants, 27.5% (378) were offered an HIV test by the provider. 3.1% (43) participants were not included in the regression analysis for whom it was unknown if the provider offered HIV testing.

^2^ Data were missing for 4 participants due to corrupted Audio Computer-Assisted Self-Interview (ACASI) entries.

^3^ Casual labourers included those reporting daily or weekly wages.

^4^ National Health Insurance Fund

^5^ Includes payments made by company medical insurance or private insurance schemes

^6^ Risk group assessed only in those who reported to be sexually active in the past six weeks (n=916)

^7^ Key populations include men who have sex with men (MSM) (n=3), sex workers (n=15) and people who inject drugs (PWID) (n=1)
