# Supplementary material for: High patient acceptability but low coverage of provider-initiated HIV testing among adult outpatients with symptoms of acute infectious illness in coastal Kenya
Source: PLoS One. 2021 Feb 5;16(2):e0246444. doi: 10.1371/journal.pone.0246444 (PMC7864413; doi:10.1371/journal.pone.0246444)
Supplement: S3 Table — (DOCX) [file pone.0246444.s003.docx]

**S3 Table. Characteristics of healthcare workers in provider focused group discussions from 6 health care facilities, coastal Kenya 2017-2019 (n=57)**

| Characteristics | N (%) |
| --- | --- |
| Gender |  |
| Female | 40 (70.2) |
| Male | 17 (29.8) |
| Age (Years) |  |
| 18 -39 | 41 (71.9) |
| >39-60 | 16 (28.1) |
| Organisation Type |  |
| Public | 39 (68.4) |
| Private | 18 (31.6) |
| Cadre |  |
| Clinical staff^1^ | 36 (63.2) |
| HIV testing counsellors | 10 (17.5) |
| Administrative staff | 8 (14.0) |
| Others2 | 3 (5.3) |
| Experience in HIV care |  |
| None | 10 (17.5) |
| < 5 years | 25 (43.9) |
| 5-10 years | 15 (26.3) |
| > 10 Years | 7 (12.3) |
| Clinical staff included medical and clinical officers, nurses, laboratory and pharmaceutical technologists.  ^2^ Other staff included nutritionists, public health officers and mentor-mothers. | |
